# Prenatal listening to songs composed for pregnancy and symptoms of anxiety and depression: a pilot study

**DOI:** 10.1186/s12906-017-1759-3

**Published:** 2017-05-08

**Authors:** Chineze Nwebube, Vivette Glover, Lauren Stewart

**Affiliations:** 1 Department of Psychology, Goldsmiths, University of London, 8 Lewisham Way, London, SE14 6NW UK; 2 Institute of Reproductive and Developmental Biology, Imperial College London, Hammersmith Campus, Du Cane Road, London, W12 0NN UK; 3 Centre for Music in the Brain, Department of Clinical Medicine, Aarhus University & The Royal Academy of Music Aarhus/Aalborg, Nørrebrogade 44, 8000 Aarhus C, Denmark

**Keywords:** Prenatal, Anxiety, Depression, Music

## Abstract

**Background:**

Prenatal anxiety and depression are distressing for the expectant mother and can have adverse effects on her fetus and subsequently, her child. This study aimed to determine whether listening to specially composed songs would be an effective intervention for reducing symptoms of prenatal anxiety and depression.

**Methods:**

Pregnant women were recruited online and randomly assigned to one of two groups: the music group (daily listening to specially composed songs) or control group (daily relaxation) for 12 weeks each. Self-report questionnaires were used to assess symptoms of State and Trait anxiety (Spielberger) and depression (Edinburgh Postnatal Depression Scale (EPDS)). Trait anxiety was measured as the primary outcome, while State anxiety and depression were the secondary outcomes. 111 participants were randomised to each group. 20 participants in the intervention group and 16 participants in the active control group completed the study.

**Results:**

The music group demonstrated lower Trait Anxiety (*p* = .0001) (effect size 0.80), State Anxiety (*p* = .02) (effect size 0.64), and EPDS (*p* = .002) (effect size 0.92) scores at week 12 compared to baseline, by paired t test. There were no such changes in the control group.

**Conclusions:**

Though this pilot study had high levels of attrition, the results do suggest that regular listening to relaxing music should be explored further as an effective non-pharmacological means for reducing prenatal anxiety and depression.

**Trial registration:**

ClinicalTrials.gov NCT02776293 LV-001. Registered 17 May 2016. Retrospectively registered.

**Electronic supplementary material:**

The online version of this article (doi:10.1186/s12906-017-1759-3) contains supplementary material, which is available to authorized users.

## Background

There is good evidence that if a mother is anxious, depressed or stressed while she is pregnant, this is not only distressing for the mother, but it can also cause problems for her future child [1]. The child is at increased risk for anxiety and depression, behavioural problems such as Attention Deficit Hyperactivity Disorder (ADHD) and conduct disorder, as well as sub-optimal cognitive development [2]. There is also an increased risk of physical problems such as asthma and altered immune function [3, 4]. All this appears to be, at least in part, due to fetal programming. The mother is also more likely to suffer from postnatal depression if she is anxious or depressed while pregnant and her baby is likely to be premature and smaller for gestational age [5]. These consequences can occur with mild to moderate symptoms of anxiety and depression, not only with severe stress or a clinically diagnosed mental illness [6]. It is therefore very important both to detect and to help ameliorate symptoms of anxiety and depression in pregnant women [7]. Many women do not want pharmacological interventions at this time, and with less severe symptoms it may not be appropriate. Thus there is a need to find non-pharmacological methods of intervention. Psychological therapies such as cognitive behavioural therapy or interpersonal therapy can be effective but these are often not available for the large numbers of women who could benefit.

There is a large body of research showing that listening to music can alter mood and arousal [8]. In terms of clinical applications, it can, for instance, lessen anxiety before dentistry [9] and reduce anxiety and depression in older adults [10]. A study with pregnant women who listened to music prior to amniocentesis showed that it reduced both state anxiety and cortisol levels [11], while an intervention that involved pregnant women learning to sing lullabies during pregnancy was found to aid emotional expression, reduce the amount of self-reported anxiety and contribute to a positive experience during pregnancy [12]. Interestingly, animal studies have shown that exposure to music during pregnancy can benefit brain development in the offspring. Chikahisa et al. [13] showed that if mice were played Mozart in the perinatal period it enhanced the learning performance and altered the brain Brain Derived Neurotrophic Factor/ Tropomyosin Kinase B (BDNF/TrkB) signaling in the adult offspring. Sheikhi and Saboory [14] have shown that exposure of pregnant rats to classical music throughout pregnancy resulted in the offspring having significantly higher cell density in the parietal cortex while Kim et al. [15] have shown that exposure to music during pregnancy increased neurogenesis in the motor and somatosensory cortex of the rat pups.

In this small pilot study we explored the possibility that listening to original songs specially composed for the prenatal period may reduce symptoms of anxiety and depression in pregnant women over a period of 12 weeks. We used a control condition of sitting quietly and undisturbed, which has been shown in an acute study to reduce anxiety and cortisol levels [16]. Proof of efficacy would justify investment into music as an accessible, inexpensive and non-stigmatizing method with potential to improve the mother’s mental health and benefit both the mother and child.

## Methods

The study was conducted between March 2014 and August 2015. The Ethics committee at Goldsmiths, University of London, approved this study. Informed consent was obtained online, prior to the start of the questionnaires.

### Participants

The women were recruited online via a specially designed website, The Relaxation in Pregnancy Study. The study was publicized on various websites, such as Netmums, and also by leaflets that were distributed in hospitals, ultrasound clinics, baby and toddler groups and coffee shops throughout London, UK. Pregnant women over 18 years from English speaking countries were eligible. Participants were randomized through a blocked design of random sizes using research randomizer. A research associate not involved in the study conducted the randomization. Individual group assignments were placed in opaque sealed envelopes to ensure allocation concealment.

**Fig. 1:**
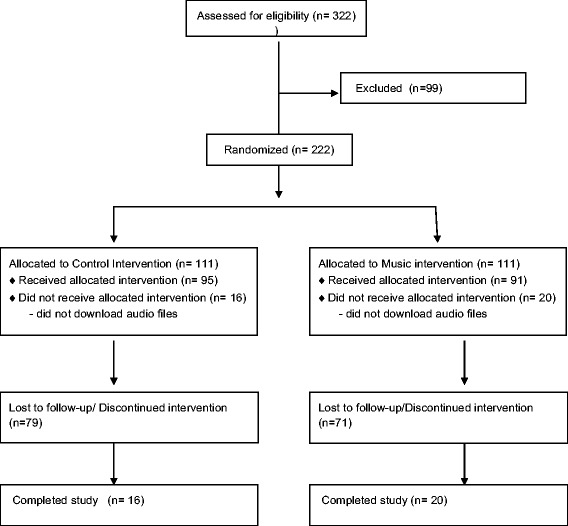
Flow diagram of participant progress through the phases of the study

Two hundred twenty-two participants completed the questionnaires on entry into the study and were randomly allocated to the control or music group (control group 111; music group 111). Their characteristics are shown in Table 1. There were no significant differences between these two groups. Thirty-six of these (control group 16; music group 20) also completed the questionnaires at 12 weeks. The attrition rate was comparable across both groups. Figure 1 illustrates the number of participants per group at each stage of the study.

**Table 1:** Baseline Characteristics of participants in control (*n* = 111) and music groups (*n* = 111)

	Total	Control	Music Group
Country, N (%)			
Australia,	1 (0.46%)	1 (100%)	0
Canada	23 (10.60%)	8 (35%)	15 (65%)
China	1 (0.46%)	0	1 (100%)
India	1 (0.46%)	0	1 (100%)
Malaysia	1 (0.46%)	0	1 (100%)
Philippines	1 (0.46%)	0	1 (100%)
Romania	1 (0.46%)	1 (100%)	0
United Kingdom	187 (86%)	96 (51%)	91 (49%)
United States	1 (0.46%)	0	1 (100%)
Gestational Age	15.84	15.37 (8.74)	16.29 (8.88)
Age Finished Full Time Education, N (%)			
Over 25	30 (18%)	13 (43%)	17 (57%)
Under 25	59 (36%)	32 (54%)	27 (46%)
Under 22	71 (43%)	32 (45%)	39 (55%)
Under 16	4 (3%)	1 (25%)	3 (75%)
Method of Recruitment, N (%)			
Conference	2 (1.8%)	1 (50%)	1 (50%)
Family Member	1 (0.9%)	0	1 (100%)
Flyer	1 (0.9%)	1 (100%)	0
Friend	5 (4.6%)	2 (40%)	3 (60%)
Website	14 (12.8%)	7 (50%)	7 (50%)
Mother & Baby	1 (0.9%)	1 (100%)	0
Mumsnet	8 (7.3%)	3 (37.5%)	5 (62.5%)
NCT	2 (1.8%)	2 (100%)	0
Netmums	60 (55%)	31 (52%)	29 (48%)
Physician	2 (1.8%)	1 (50%)	1 (50%)
Recruiter	4 (1.8%)	0	4 (100%)
Social Media	9 (8.3%)	4 (44%)	5 (56%)
Psychometric Characteristics			
Trait Anxiety (Spielberger)	41.02 (12.28)	42.39 (11.13)	39.75 (13.26)
State Anxiety (Spielberger)	39.15 (13.46)	40.15 (13.15)	38.23 (13.83)
Depression (EPDS)	8.75 (6.14)	9.85 (6.77)	7.73 (5.36)

Sample size estimations have been made to power the study using G*Power 3.1. An effect size of 0.57 was estimated based on effect sizes of studies involving forms of relaxation on Trait Anxiety [17]. Assuming an effect size of 0.57, and a two-tailed alpha of 0.05, a sample of 50 participants per group was deemed necessary to generate a power of 0.80.

Both the control and music groups were asked to listen to their assigned audio file for at least 20 min a day and to record each time they had engaged in this activity. Twelve weeks after each participant had started the study they were prompted to complete the EPDS, and State-Trait Anxiety Index for a second time. Participants were sent follow- up emails every 2 weeks that served as a reminder for the participants to listen to their assigned mp3 files. At the end of 12 weeks, participants were prompted to complete the questionnaire. Participants who had not completed their questionnaire by the deadline were prompted to complete the questionnaire every 5 days past the deadline for a maximum of 3 times.

### Materials

Qualtrics software was used to administer the questionnaires and participants were prompted to complete these via email at the respective time points. Participants received an audio file specific to the assigned group electronically. Both audio files began with a 2 min introduction given via the same speaker; following this, participants were instructed to either sit quietly and undisturbed (control group) or to listen to pre-recorded songs (music group), for 20 min. Composer Jennie Muskett wrote these songs specifically for use during pregnancy. The songs were composed using specific tempos, musical shapes and phrases aimed at inducing a calm state. The music was original and therefore had no previous associations. Use of some of the musical characteristics typical of traditional lullabies, incremental reductions in tempo, and repetition of musical phrases, many of which were derived from motherese, all aimed to further enhance the effect. The song lyrics invited connection with, and visualisation of the fetus and each song addressed various issues relevant to pregnant women. The instrumentation and timbre was intimate, performed vocally and accompanied entirely by acoustic instruments.

### Self-report questionnaires

Symptoms of Trait anxiety, the primary outcome, and symptoms of State Anxiety and Depression, the secondary outcomes, were assessed using self-report questionnaires.

#### Edinburgh postnatal depression scale

The Edinburgh Postnatal Depression Scale [(EPDS), validated for prenatal use] is a 10 item questionnaire measuring symptoms of maternal depression. This scale is scored from 0 to a maximum of 30, with a higher number representing a more depressed state [18].

#### State - trait anxiety inventory

The State – Trait Anxiety Inventory is a questionnaire widely used for measuring maternal anxiety [19]. The index is divided into state and trait, each composed of 20 items on a 4 point likert scale. Answer choices range from 1 (not at all) to 4 (very much so). The questionnaire is scored from 20 to a maximum of 80 with a higher number representing a more anxious state. The Spielberger State Index measures the anxiety of an individual at the time of taking the questionnaire whereas the Spielberger Trait Index measures an individual’s general level of anxiety.

## Results

There were no significant differences in baseline gestational age in weeks (mean ± SD) at baseline (13.7 ± 9.0 versus 18.2 ± 7.7) or days spent relaxing or listening (38.8 ± 21 versus 44.3 ± 23.7) between the control (*n* = 16) and music group (*n* = 20) respectively, who completed the study.

Table 2 shows the baseline and 12-week scores for symptoms of anxiety (Trait and State) and symptoms of depression (EPDS). Paired t-tests demonstrated significantly lower scores in the music group at week 12 compared to baseline in all 3 measures. The effect sizes for Trait anxiety, State anxiety, and Depression were 0.80, 0.64, and 0.92, respectively. In the control group, no significant differences were found [See Additional file 1 for complete dataset].

**Table 2:** Anxiety and depression scores at baseline and 12 weeks in the control (*n* = 16) and music groups (*n* = 20) who completed the study

	Control baseline	Control 12 weeks	*p*	Music baseline	Music 12 weeks	*p*
Spielberger Trait	42.8 ± 8.0	38.8 ± 12.5	0.155	38.5 ± 10.9	30.6 ± 8.6	0.001
Spielberger State	38.9 ± 12.0	35.2 ± 14.3	0.138	37.1 ± 12.1	30.3 ± 8.9	0.02
EPDS	9.3 ± 5.1	7.3 ± 6.0	0.095	8.5 ± 5.3	4.0 ± 4.4	0.002

Comparison in Scores by paired t-test. *P* value tests for significant differences between the two time points

## Discussion

The results of this small study look promising, in that over the 12-week period, participants’ symptoms of anxiety and depression decreased significantly in the music group and did not change significantly in the control group. It is of note that the baseline scores in both groups are above average [19]; it appears that women with somewhat raised symptoms of anxiety and depression chose to take part in the study.

There are several obvious limitations to this study. The first is the high attrition rate. This may well be because the investigators had no direct contact with the participants, as all recruitment and follow up was online. It may also be that only some women find listening to music helpful or enjoyable; but an intervention that benefits a subgroup could still be of great value. Though only the music group showed significant reduction in anxiety and depression, the interaction between group and time was not statistically significant using a Mixed Analysis of Variance (ANOVA). This is likely due to the small numbers, and emphasizes the need for a larger study, with personal contact and better retention. We also do not know if other music would be equally effective. The songs used here were specifically composed and designed to have a calming effect.

Despite these limitations, the magnitude of reduction of the scores in the music listening group is notable, from a mean of 38.5 to 30.6 for Trait anxiety and from a mean of 8.4 to 3.75 for the EPDS. We know that the means of the scores for these questionnaires generally tend to remain quite stable over pregnancy [20], and reductions of this magnitude could be clinically significant.

## Conclusions

These results do suggest that further research in this area is warranted. Listening to music during pregnancy is a potentially enjoyable, inexpensive and non-stigmatizing intervention that may also be beneficial for the future child.

## Additional file

Additional file 1: Dataset: EPDS, State Anxiety and Trait Anxiety Scores at Baseline and 12 weeks in the control and music groups.

## Abbreviations

ADHD: Attention deficit hyperactivity disorder
ANOVA: Analysis of variance
BDNF: Brain derived neurotrophic factor
EPDS: Edinburgh Postnatal Depression Scale
TrkB: Tropomyosin receptor kinase B
